# Supplementary material for: Preparation of Magnetic MIL-68(Ga) Metal–Organic Framework and Heavy Metal Ion Removal Application
Source: Molecules. 2022 May 26;27(11):3443. doi: 10.3390/molecules27113443 (PMC9182009; doi:10.3390/molecules27113443)
Supplement: Supplementary file 1 [file molecules-27-03443-s001.zip › molecules-1734530-supplementary.pdf]

Preparation of magnetic MIL-68(Ga) metal-organic framework and heavy metal ion removal application

Youjun Zhang [1,3], Licong Liu [4], Dixiong Yu [2], Jinglan Liu [3], Lin Zhao [3], Jinpeng Liu [5], and Shuo Liu [2,5]*

[1] School of Environmental Science and Engineering, Tianjin University, 92, Weijin Rd., Nankai District, 300350, China; zhangyoujun001@126.com
[2] Key Laboratory of Molecular Microbiology and Technology, Ministry of Education, Department of Microbiology, College of Life Sciences, Nankai University, Tianjin 300071, China
[3] Tianjin North China Geological Exploration Bureau，67, Guang-rui-xi-lu Rd., Tianjin, 300170, China
[4] Technical Centre of Solid Waste and Soil Ecology of Inner Mongolia, Teng-fei Rd., Huhehaote, 010000, China
[5] College of Environmental Science and Engineering, Tianjin Key Laboratory of Environmental Remediation and Pollution Control, Nankai University, 38 Tongyan Rd., Tianjin 300350, P. R. China

* Correspondence: liushuo@nankai.edu.cn

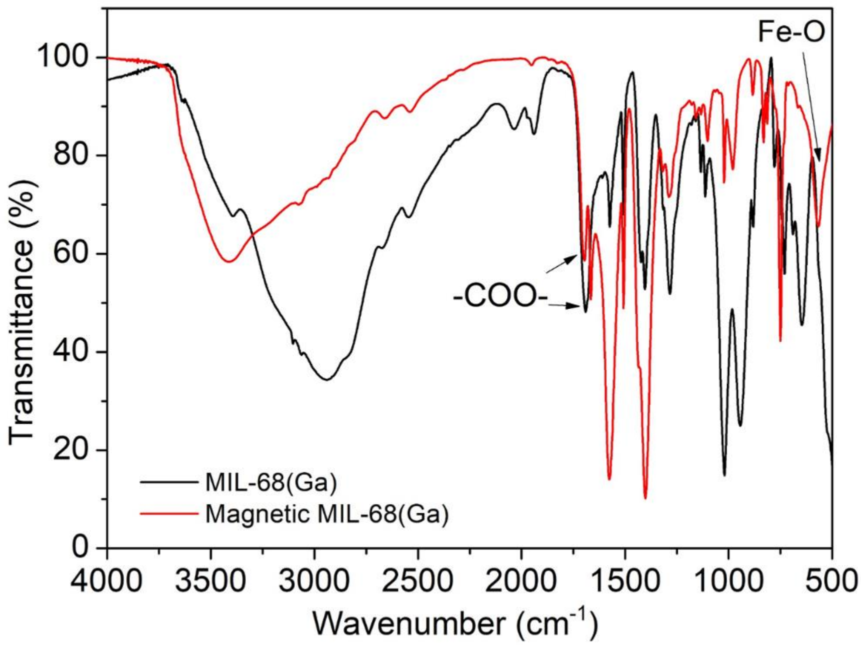

Figure S1. FT-IR spectrum of MIL-68(Ga) and magnetic MIL-68(Ga).

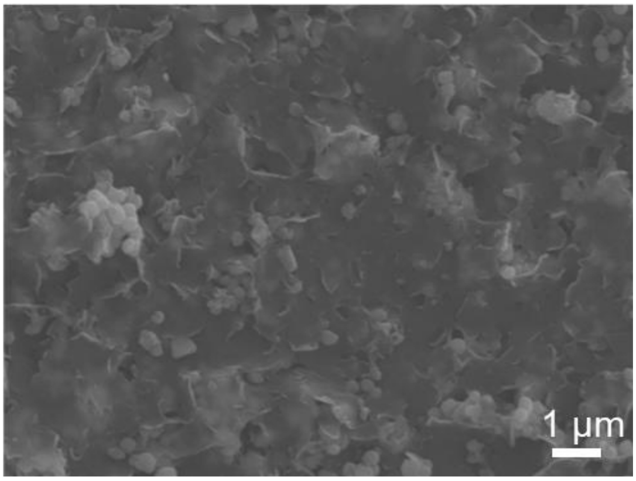

Figure S2. SEM images of magnetic MIL-68(Ga) after incubation in acidic solution (pH = 5) for 30 min.

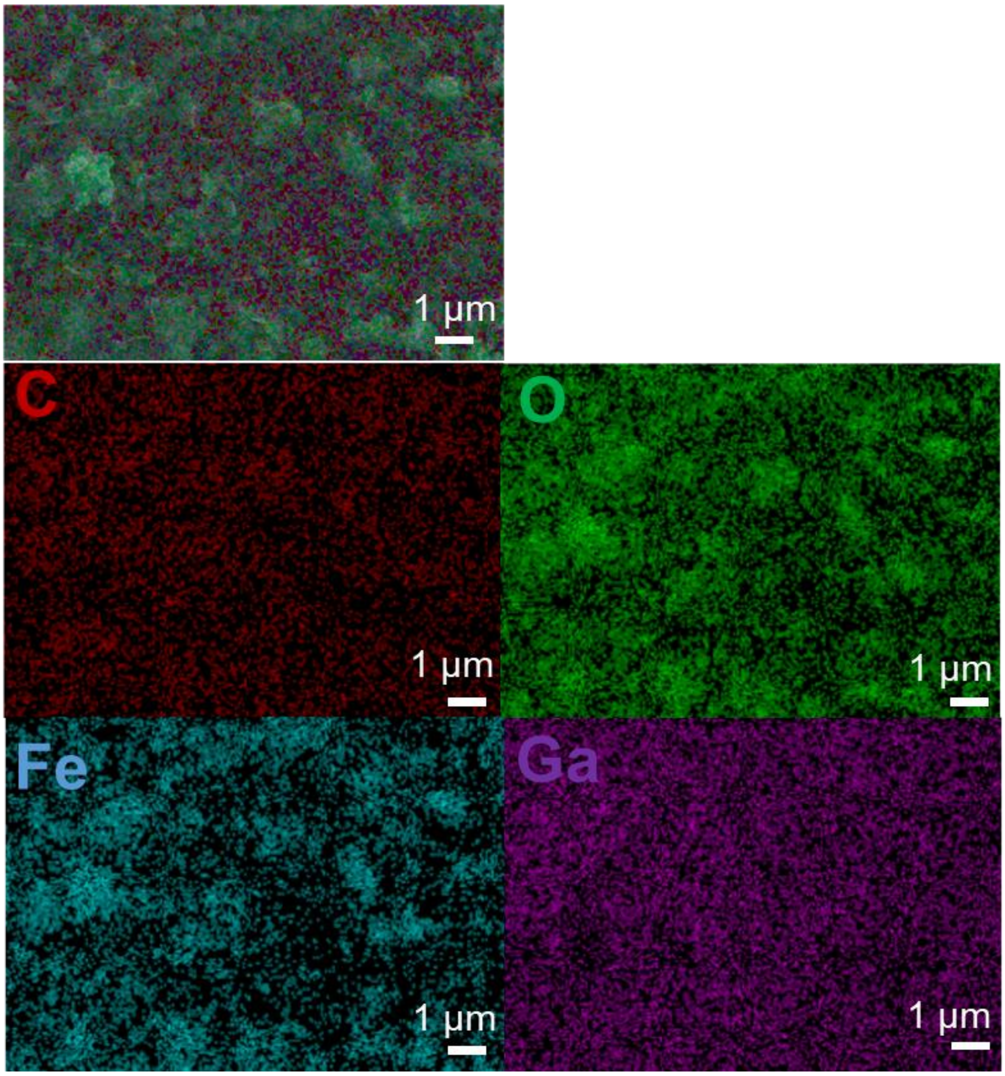

Figure S3. EDS mapping images of magnetic MIL-68(Ga) after incubation in acidic solution (pH = 5) for 30 min.

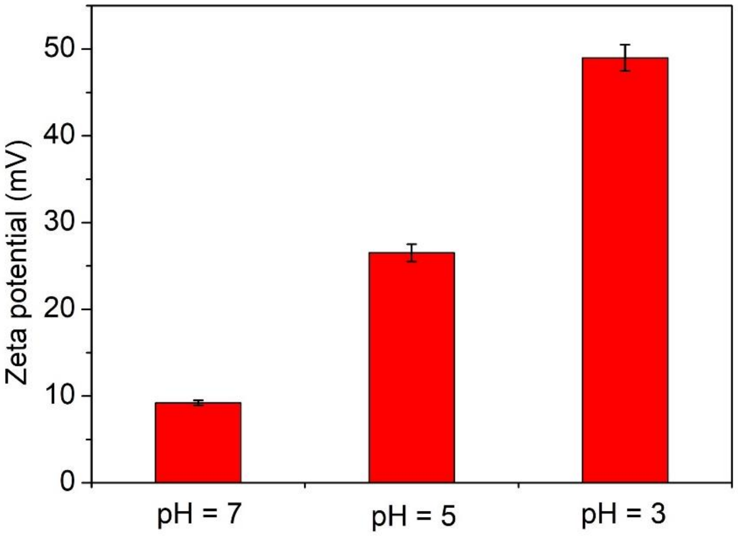

Figure S4. Effect of pH on the zeta potential of magnetic MIL-68(Ga).

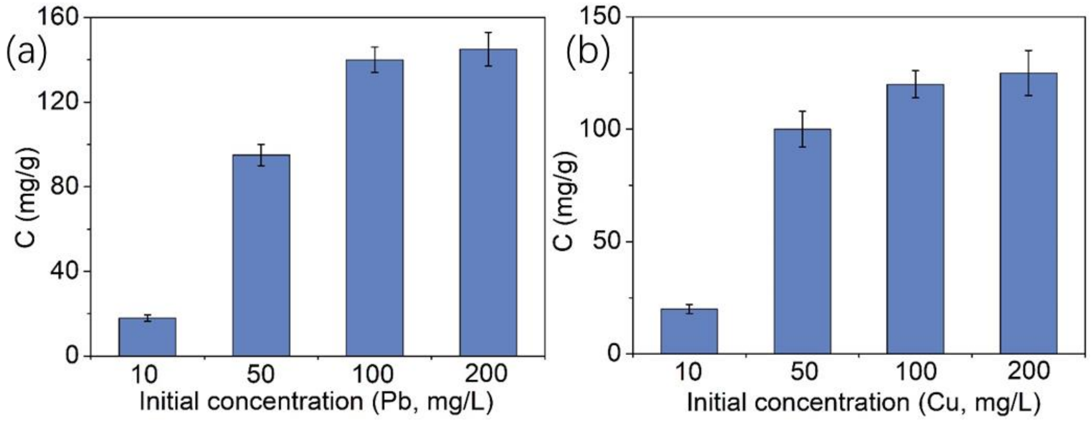

Figure S5. Adsorption capacity of MIL-68(Ga) on Pb^{2+} (a) and Cu^{2+} (b). (50 mg/L of metal ions, 200 mg/mL of adsorbent, 1 mL solution, pH = 7, room temperature, 30 min of incubation time).

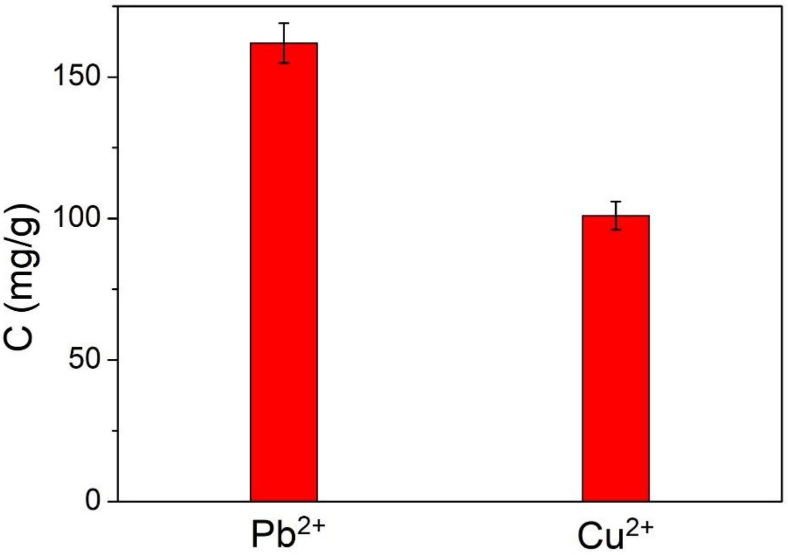

Figure S6. Adsorption capacity of MIL-68(Ga) in the mixture of Pb^{2+} and Cu^{2+} (100 mg/L of Pb^{2+} and Cu^{2+}, 200 mg/mL of adsorbent, 1 mL solution, pH = 7, room temperature, 30 min of incubation time).

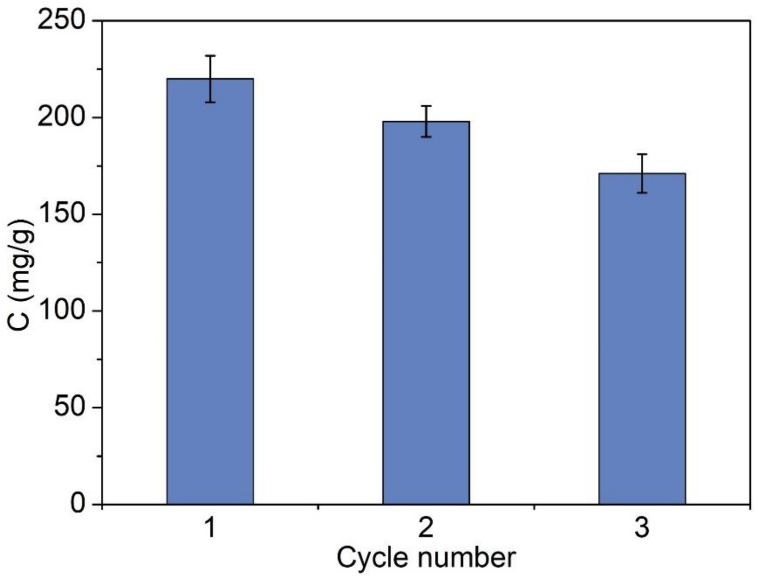

Figure S7. Reusability evaluation of magnetic MIL-68(Ga) (50 mg/L of metal ions, 200 mg/mL of adsorbent, 1 mL solution, pH = 7, room temperature, 30 min of incubation time).
